# *Aeromonas* species endogenous endophthalmitis

**DOI:** 10.1099/jmmcr.0.005094

**Published:** 2017-05-04

**Authors:** Laura Ryan, Gareth Higgins, Maeve Doyle

**Affiliations:** ^1^​ Department of Microbiology, University Hospital Waterford, Waterford, Ireland; ^2^​ Department of Ophthalmology, University Hospital Waterford, Waterford, Ireland

**Keywords:** aeromonas, bloodstream infection, gastroenteritis, endogenous endophthalmitis, enucleation

## Abstract

**Introduction:**

*Aeromonas* spp. are Gram-negative bacteria classically associated with water sources and a variety of clinical infections in humans.

**Case presentation:**

A 79-year-old female patient presented with gastroenteritis with associated *Aeromonas* spp. bloodstream infection. Two days after admission she developed eye symptoms and was diagnosed with endophthalmitis and underwent emergency evisceration and implant. *Aeromonas* spp. was also recovered from intra-ocular samples.

**Conclusion:**

In this case gastroenteritis caused by *Aeromonas* spp. was complicated by bloodstream infection which seeded to the eye, resulting in rapidly progressive endogenous endophthalmitis.

## Abbreviations

API, analytical profile index; CRP, C-reactive protein; CT, computed tomography; h, hours; MALDI-TOF MS, matrix assisted laser disorption/ionisation time of flight mass spectrometry; spp, species; TB, tuberculosis; WCC, white cell count.

## Introduction

Endophthalmitis is bacterial or fungal infection inside the eye involving the vitreous and/or aqueous humors. Most frequently it is exogenous, occurring after eye surgery, penetrating ocular trauma or as an extension of corneal infection. Endogenous endophthalmitis, bacterial or fungal seeding of the eye, is a challenging and devastating complication of bacteraemia, and accounts for approximately 6 % of all cases of endophthalmitis [1].

Pathogens implicated in endogenous endophthalmitis vary by country. In western countries the predominant pathogens include: *Staphylococcus aureus* (25 %); *Streptococcus* spp. (30–50 %); and Gram-negative bacilli (30 %). In Asia, the majority of cases are associated with *Klebsiella pneumoniae* related to liver abscesses [2]. Worldwide, Gram-negative infections (55 %) are more common than Gram-positive (45 %), especially in Asia, and 24 % require evisceration or enucleation [3].

Endogenous endophthalmitis is associated with a positive blood culture in approximately half of the cases and is frequently associated with Gram-negative bacteria such as *Escherichia coli* from urinary or intra-abdominal sources [3].

A published review of endogenous endophthalmitis from 1986 to 2012 found that 60 % of patients had a predisposing condition, most commonly diabetes, intravenous drug use and malignancy [3]. Most common sources include liver, lung, endocardium, urinary tract and meninges [3].

The genus *Aeromonas* consists of Gram-negative bacilli which are oxidase-positive, facultative anaerobes and ubiquitous in nature, especially in aquatic environments. Species from this genus have been implicated in many infections in humans including wound infections, cellulitis, necrotising fasciitis, gastroenteritis, intra-abdominal infections and dissemination to meninges or endocardium. Infections have been reported in immunocompetent as well as immunocompromised patients [4].

One study in Bangladesh found a carriage rate of *Aeromonas* spp. in healthy children of 3.3 % (27/830), with an isolation rate from faeces of 7.2 % (125/1735) in diarrhoeal children [5]. Other published studies found similar rates [6]. *Aeromonas* spp. bacteraemia secondary to gastroenteritis has been documented [7].

Bloodstream infection with *Candida* spp. prompts routine examination for endophthalmitis as recommended in current international guidelines [8], yet other organisms frequently isolated in blood cultures may also seed to the vitreous and/or aqueous humors but awareness of this may be lacking.

Treatment of the underlying source of bacteraemia with systemic antibiotics is necessary but will not always effectively treat the endophthalmitis. Intravitreal antibiotics and vitrectomy may be necessary [2].

## Case report

A 79 year female presented with confusion, low grade temperature and diarrhoea and vomiting for 24 h, which had resolved at time of presentation to the emergency department. She also had right eye itch which had progressed to erythema and pus, with the eyelid being ‘stuck-down’, there was no pain. This was felt to be conjunctivitis by admitting team and an ophthalmology referral was made.

On further questioning of family members, the patient had had vomiting and diarrhoea for less than 24 hours which had resolved 48 h prior to admission. She had complained of not being able to see from her right eye 24 h prior to admission. Development of confusion then prompted review by her general practitioner (GP) and admission to hospital. Her background history included a nephrectomy at age 34 for TB, atrial fibrillation, cholecystectomy and bilateral total hip replacements.

## Investigations

On admission:

C-reactive protein (CRP) 257.1 mg l^−1^.

White cell count (WCC) 18.1×10^9^ l^−1^.

Neutrophil count 16.53×10^9^ l^−1^.

Blood cultures (two sets) taken on admission became positive at approximately 9 h and small Gram-negative bacilli were seen on Gram staining. Empiric amoxicillin–clavulanate was changed to piperacillin–tazobactam and ciprofloxacin pending identification and susceptibility testing.

Ophthalmology review found ‘kissing’ choroidal haemorrhage with an obliterated anterior chamber secondary to anticoagulation therapy and recent vomiting.

The blood culture isolate was identified by MALDI-TOF MS as a species of the genus *Aeromonas* on two-hour growth and the antibiotic regime was changed to meropenem. No species-level identification was given for this sample.

CT brain showed bilateral small vessel ischaemia but no acute intracranial haemorrhage to account for confusion. The CRP was now 178.0 and WCC 16.7 and she was apyrexial.

The isolate was found to be resistant to amoxicillin and amoxicillin–clavulanate, but susceptible to ciprofloxacin, gentamicin, cefotaxime, piperacillin–tazobactam and meropenem. The antimicrobial therapy was rationalised to ceftriaxone and ciprofloxacin, with penetration of eye tissue in mind.

A species of the genus *Aeromonas* was isolated from intraocular specimens from the theatre. API 20NE identified the isolate as *Aeromonas sobria*, while MALDI-TOF MS identified it as *Aeromonas vilnerii*, conflicting species level identifications according to the two methods employed on this sample.

Blood cultures taken 48 h after admission, and multiple cultures thereafter, were sterile.

Initial eye swabs culture on admission: diphtheroids and coagulase negative staphylococci.

Faeces sample on admission was negative (EntericBio).

Trans-thoracic echocardiogram was inconclusive with regards to whether the mitral valve was normal or not. Trans-oesophageal echocardiogram was not performed, as per the patient’s wishes while she was lucid.

CT thorax abdomen and pelvis showed no evidence of seeding to viscera.

## Diagnosis and treatment

At ophthalmology follow-up, the patient commented on brown liquid coming out of her right eye in the previous hour. On examination there was a corneal perforation with brown purulent discharge and no fundal view. Endogenous endophthalmitis was diagnosed and she was brought to operating theatre for corneal glueing, vitreous and aqueous tap and intravitreal antibiotics under local anaesthesia. In theatre the cornea was found to be almost completely destroyed with prolapse of uveal tissue. The procedure was abandoned and arrangement made for theatre the following day, when the patient underwent evisceration and hydroxyapatite orbital implant the following day under general anaesthetic.

On day two post-operatively her CRP and WCC continued to fall, 64.9 and 10.8 respectively, but she remained confused.

## Outcome and follow-up

It came to light in discussion with family members later in admission the patient had been drinking water from the hot water storage tank, as the cold water tap had not been working, this may have been the source of acquisition of infection. Unfortunately this water was not sampled for culture or assessed for temperature.

Two weeks post evisceration, she had a non-ST elevation myocardial infarction and continued to be confused and became agitated. She developed heart failure and in discussion with family members it was decided not to resuscitate in the event of a cardiopulmonary arrest.

Her heart failure progressed and one week later, comfort measures were put in place and antimicrobials were discontinued.

## Discussion

*Aeromonas* spp. is highlighted here as an aggressive and destructive organism capable of seeding to distant sites from bacteraemia, even in immunocompetent hosts. There has been a report of endogenous endophthalmitis caused by *Aeromonas hydrophila* in an immunocompetent patient in Japan [9].

*Aeromonas* spp. are infrequently reported as a cause of endophthalmitis – sources include biliary, intra-abdominal and unknown in an immunocompromised patient, as well as penetrating wounds [9–12]. There are very few cases reported, however, a common feature of all of them is the associated rapid clinical course and poor prognosis [9–11]. Unfortunately the source of acquisition of *Aeromonas* in this case is unclear, but consumption of hot water from the storage tank is one potential source.

This case also shows the difficulty in identifying isolates of the genus *Aeromonas* to species level. MALDI-TOF MS has now obviated the need for many of the phenotypic tests traditionally used in identification of species of the genus *Aeromonas*. It is a Gram-negative facultative anaerobe, catalase- and oxidase-positive, non-sporing and motile. One important distinguishing quality is the inability to grow in the presence of 6.5 % sodium chloride. Isolates grow optimally at temperatures of 22–35 °C but some may grow at temperatures of 0–45 °C. Species level identification is difficult as there is extensive phenotypic diversity within the genus [13].

Important learning points from this case are that bloodstream infections with Gram-negative bacteria can seed to the globe and cause endophthalmitis and that prompt recognition and treatment of this infection are essential.
